# Preoperative risk factor analysis of postoperative stroke after Cox-maze procedure with mitral valve repair

**DOI:** 10.1186/1471-2261-14-116

**Published:** 2014-09-11

**Authors:** Jun Seok Kim, Song Am Lee, Jae Bum Park, Hyun Keun Chee, Jin Woo Chung

**Affiliations:** Department of Thoracic and Cardiovascular Surgery, Konkuk University Medical Center, Konkuk University, School of Medicine, 120-1 Neungdong-ro Hwayang-dong, Gwangjin-gu, Seoul 143-729 Korea

**Keywords:** Atrial fibrillation, Stroke

## Abstract

**Background:**

Atrial fibrillation (AF) is a life-threatening arrhythmia that carries the high risk of thromboembolic complication. Stroke often develops in patients who undergo successful Cox Maze procedure, despite the fact that the procedure has shown a high rate of success in sinus conversion from AF. This study examined the preoperative risk factors predictive of stroke following Cox Maze procedure in patients with mitral valve disease.

**Methods:**

240 patients with the mean age of 57 years underwent Cox-Maze IV procedure with mitral valve repair from November 2007 through December 2010. All patients were available during the follow-up period with the mean duration of 23.6 months. This study excluded those patients who had undergone mitral valve replacement because of maintenance of warfarin medication

**Results:**

Sixteen patients had an ischemic stroke. Of these sixteen patients, six had a transitional ischemic accident while the remaining ten had cerebral infarction. Twelve of sixteen showed sustained sinus rhythm, three showed AF and one had pacing rhythm. Univariate analysis showed that only preoperative stroke history was associated with postoperative stroke (p = 0.03). High CHA_2_DS_2_-VASc score, rheumatic etiology, large left atrium (LA), preoperative or postoperative LA thrombus, age, sex, hypertension, and concomitant surgery were not associated with predictive risks for stroke.

**Conclusions:**

In the group of patients who underwent the Cox-Maze procedure with mitral valve repair, having a stroke history was the only preoperative risk factor that could lead to a stroke event after surgery. Accordingly, patients with affliction of ischemic stroke, albeit sustained sinus rhythm, may require prophylactic anticoagulation.

## Background

Atrial fibrillation (AF) is the most significant arrhythmia that may cause thromboembolic complications. AF under any circumstance causes reduction of cardiac output and thrombus formation that may provide the grounds for systemic embolism. AF increases the risk of death by nearly twice and ischemic stroke by about five folds [1]. In particular, AF occurs in 30~50% from the group of patients with mitral valve disease [2, 3]. With respect to the reason for a hospital visit in these patients, there are more cases with symptoms associated with stroke than there are cases with symptoms related to cardiac disease. AF would not largely disappear by surgical modality with mitral valve repair alone in such group of patients.

Advances in the Cox-Maze procedure have made it the most valid method in AF treatment. Its complex techniques have continuously been modified and advanced [4] to the point of achieving 90% success rate in surgery [5–7].

The success rate of having sinus conversion of the heart rhythm after Cox-Maze procedure is considerable in patients with a mitral valve disease. Nevertheless, ischemic stroke occurs quite often in patients with successful sinus conversion. The reason may be that patients have either transient reversion to AF or a cerebral arterial disease. However, a recent study revealed that the incidence rate of postsurgical stroke had been high in patients with a large left atrium devoid of contractile function in the left atrium [8].

This study examined the incidence rate of stroke following mitral valve surgery and Cox-Maze procedure in AF patients with mitral valve disease. This study retrospectively analyzed various preoperative parameters and investigated risk factors that could cause stroke after surgery.

## Methods

### Patients

This study included 240 patients who had been diagnosed as having mitral valve disease and AF from November 2007 through December 2010. The mean age was 56.9 years and the male proportion was 38.0%. Demographic data was summarized in Table 1. All of these patients underwent Cox Maze procedure and mitral valve repair. Because of continuous postsurgical warfarin administration for anticoagulation therapy, those patients who had undergone mitral valve replacement were excluded. AF was confirmed in the 12-lead electrocardiogram performed for all patients within a week prior to surgery. All data of medical records, clinical history, electrocardiogram and echocardiography were retrospectively collected after surgery, and the data were accounted for all patients while follow-up observations were attainable for all patients. The Guidelines for Reporting Data and Outcomes for the Surgical Treatment of Atrial Fibrillation [9] were used for the definition of AF recurrence, data analysis and comparison. The Institutional Review Board at Konkuk University Medical Center approved this study (No. KUH1080013).

**Table 1:** **Demographic data and operative profiles**

Number of patients	240
Age, years	56.9 ± 12.1
M/F, n	91/149
HTN, n (%)	39 (16.3)
History of preoperative stroke	28 (11.7)
Prior cardiac surgery, n (%)	8 (3.3)
AF type	
Paroxysmal, n (%)	5 (2.1)
Persistent, n (%)	235 (97.9)
Mitral diagnosis	
Rheumatic, n (%)	139 (57.9)
Degenerative, n (%)	94 (39.2)
Others, n (%)	7 (2.9)
Presence of left atrial thrombus, n (%)	22 (9.2)
Echocardiographic data	
LV EF,%	59.7 ± 7.4
LA dimension, mm	51.0 ± 11.7
MV operation	
MV repair, n (%)	240 (100)
Concomitant cardiac surgery	
AV repair, n (%)	80 (33.3)
TV repair, n (%)	64 (26.7)
CABG, n (%)	8 (3.3)
ASD or PFO closure, n (%)	9 (3.8)
Other, n (%)	3 (1.3)

*Abbreviations*: HTN, hypertension; AF, atrial fibrillation; LV, left ventricle; EF, ejection fraction; LA, left atrium; MV mitral valve; AV, aortic valve; TV, tricuspid valve; CABG, coronary artery bypass graft; ASD, atrial septal defect; PFO, patent foramen ovale.

### Cryothermy procedure

**Figure 1:**
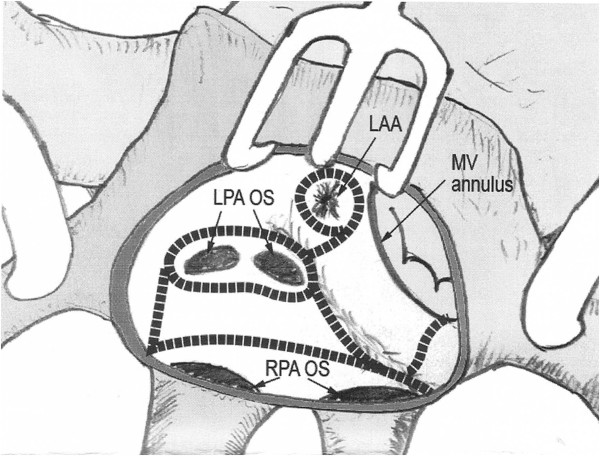
**Diagram of the left atrial Cox-Maze procedure using cryothermy energy (black dotted lines).**

The cryothermy procedure was carried out by using the modified left-sided Cox Maze IV procedure [10]. Figure 1 shows our procedure sets. After left atriotomy, separate two box lesions for pulmonary isolation were made with extended left atriotomy incision. Then, the lines between two box lesions were ablated. A line form the pulmonary isolation lesion to left atrial appendage and another line from the pulmonary isolation lesion to mitral valve annulus were made. Left atrial appendage isolation was done. With respect to deployment of cryothermy energy, cyroablation using the argon gas-based device (Medtronic Co. USA) set at -180°C was performed for one minute to obtain transmural lesion. Left atrial appendage was neither ligated nor resected.

### Surgical procedure

All surgeries were carried out under cardiopulmonary bypass after reaching moderate hypothermia and cardioplegia-induced cardiac arrest by infusing a cardioplegic solution. An antegrade approach was only used for cardioplegic solution in isolated mitral valve surgery, while a retrograde approach was used in mitral valve surgery with concomitant aortic valve surgery. Generally, the cardioplegic solution was infused every 35 minutes. Intraoperative transesophageal echocardiogram was inserted for all patients. The mitral valve was exposed by making incision on the interatrial groove for all patients, and cryothermy ablation was performed using the left-sided Cox-Maze IV procedure (Figure 1). In cases of mitral regurgitation, patch obliteration technique [11] for posterior leaflet prolapsed and artificial chordae formation techniques for anterior leaflet prolapse were employed. Annuloplasty was performed by using the Mitra-strip (ScienCity, Inc. Seoul, Korea), which had been newly developed for the posterior supraannular plication [12].

For the repair of rheumatic mitral disease, in which mitral stenosis due to leaflet calcification would be the primary lesion, 1) thickened calcified leaflet was removed by slicing, then, 2) commissurotomy was performed, followed by 3) making extension of the posterior leaflet by using a bovine pericardial patch, and then 4) annuloplasty incorporating the Mitra-strip was carried out.

The concomitant procedures included tricuspid valve repair, aortic valve repair, coronary artery bypass grafting, ASD closure or others.

### Postoperative management

Overall postoperative care was similar to that of patients with routine cardiac surgery. Amiodarone was infused to all patients except for patients who developed bradycardia (pulse rate < 60beats/min). A saturation dose of 300 mg adiodarone was slowly injected after surgery. Thereafter, an oral dose of 200 ~ 400 mg/day was administered after extubation. Amiodarone was prescribed for all other patients who did not develop bradycardia during the hospital day, and was maintained for three months after discharge from our institute. Anticoagulation therapy was also maintained for three months after discharge in patient with successful Cox-Maze procedure due to the application of annuloplasty ring. After discharge, all patients were followed-up at the outpatient clinic once every three months on the average. On each visit at the outpatient clinic, patients underwent 12-lead ECG for check-up. Amiodarone and anticoagulation were continued for patients with AF recurrence after hospital discharge. When patients developed stroke, anticoagulation therapy along with neurology consultation was immediately started again, albeit no recurrence of AF.

### Statistical analysis

Continuous variables were expressed as the mean and standard deviation while discrete variables were expressed in frequency and percentage. The Cox proportional hazards regression model as well as univariate analyses were used to determine the risk factors. SPSS Korean version 18.0 (SPSS Inc, Chicago, IL USA) was used for the statistical process and the p values less than 0.05 were considered statistically significant.

## Results

### The characteristics of the patients

Of these two 240 patients who underwent the left-sided Cox Maze IV procedure with mitral valve repair, 91 were males, 39 had hypertension and 28 had a history of thromboembolic event before surgery. Eight patients had a history of prior heart surgery. Permanent AF type was overwhelmingly numerous with the number of patients showing 235 (97.9%). Preoperative echocardiography confirmed the left atrial enlargement suggestive of a mitral valve disease in the most patients. 22 patients (9.2%) were confirmed as having a thrombus in the left atrium during surgery (Table 1).

The primary indications for surgery and pathologic findings revealed that 94 patients had degenerative mitral valve regurgitation while 139 patients had a rheumatic disease with the evidence of mitral valve stenosis with insufficiency (Table 1).

All of 240 patients underwent mitral valve repair with Cox-Maze procedure. The most frequently performed concomitant surgery was aortic valve repair (80 cases). This was followed by 64 cases of tricuspid valve repair, eight cases of coronary artery bypass grafting and nine cases of either atrial septal defect or persistent foramen ovale closure (Table 1).

### Clinical outcomes

All of 240 patients who underwent the left-sided Cox-Maze procedure with mitral valve repair were followed-up for the mean duration of 23.6 ± 7.9 months. Among these 240, sixteen subjects (6.7%) developed thromboembolic event(s) during the follow-up period. Of these sixteen patients, six suffered with mild transitional ischemic accident (TIA) but recovered symptomatically within a day. The rest of the patients were diagnosed as having cerebral infarction by neurologists as well as brain computed tomographic scan confirmation. Eight patients fully recovered with anticoagulation and rehabilitative treatment. One patient recuperated after right middle cerebral artery thrombectomy while the remaining one patient expired from pneumonia during the neurologic treatment after development of stroke. All sixteen patients were treated in the hospital at the time of thromboembolic event, and left atrial thrombus was not found for all of them in echocardiography which was performed on admission.

The time it took to development of thromboembolic event after surgery was 227.6 ± 333.6 days on the average. The ECG results of sixteen patients on admission showed that sinus rhythm was found in twelve patients (75%), AF in three patients (18.8%) and pacing rhythm in one patient (6.3%).

### Predictive risk factors of thromboembolic events after Cox-Maze procedure

We analyzed risk factors among various preoperative variables such as age, sex, rheumatic etiology of mitral valve disease, preoperative left atrial size, history of prior cardiac surgery, hypertension, presence of left atrial thrombus, concomitant cardiac surgery and history of thromboembolic events, which could affect thromboembolic events after surgery (Table 2). Univariate analysis showed that the history of thromboembolic event was significantly related to the postoperative stroke (odds ratio [OR] 3.23, 95% confidential interval [CI] 1.12-9.26, p = 0.03). Patients with CHADs2-VAS score ≥2 had a tendency to have postoperative stroke, but it did not reach statistical significance (OR 2.60, 95% CI 0.90-7.48, p = 0.08). Other parameters that listed above revealed not related with postoperative stroke (Table 2).

**Table 2:** **Preoperative risk factors of thromboembolic events**

	Univariate	
	OR (95% CI)	p value
Age, years	1.34 (0.19-10.52)	0.75
Sex	0.92 (0.34-2.48)	0.86
Rheumatic etiology	0.63 (0.24-1.63)	0.34
Preoperative giant LA (>60 mm)	0.65 (0.15-2.87)	0.57
Hypertension	0.34 (0.05-2.58)	0.30
Concomitant cardiac surgery	1.08 (0.41-2.84)	0.88
Warfarin stop at 3 M postoperatively	0.79 (0.30-2.08)	0.63
Preoperative anticoagulation	NA	
Preoperative LA thrombus	0.65 (0.09-.4.89)	0.67
Postoperative LA thrombus	2.25 (0.51-9.93)	0.28
Preoperative stroke history	3.23 (1.12-9.26)	0.03
CHADs2-VAS score	2.60 (0.90-7.48)	0.08

*Abbreviations*: LA, left atrium; M, month; OR, odds ratio; CI, confidential interval; NA, not applicable due to zero count.

## Discussion

The present study showed that preoperative history of stroke would increase the risk of postoperative stroke after Cox-Maze procedure with mitral valve repair. Buber et al. suggested left atrial mechanical contraction and left atrial size as the postoperative risks of stroke after Cox-Maze procedure [8]. However, there were few studies that analyze the risk factors for postoperative stoke. Many studies focused on the factors influencing the outcome of Cox-Maze procedure itself [13–17], but few studies focused on postoperative stroke [8]. Owing to the fact that AF increases stroke incidence by five folds [1], previous studies have focused on either restoring sinus rhythm or analysis of the risk factors that lead to failure of Cox-Maze procedure. Furthermore, the incidence of postoperative stroke is a mere 1-3% [13, 18, 19], making it difficult to analyze the risk factors of postoperative stroke. On the other hand, having the incidence rate of postoperative stroke of 6.7% in this study is considerably higher than that of other previous studies. However, heterogeneous population, witnessed in this investigation, involving a large numbers of rheumatic mitral valve and double valve diseases, may affect the incidence rate of stroke. It was remarkable that twelve of sixteen patients (75%) with postoperative stroke demonstrated sinus rhythm while eight of the twelve (67%) patients had left atrial mechanical contraction in echocardiography at the onset of stroke. This result did not concur with that of Buber et al. study [8]. Buber et al. reported fifteen cases of stroke (10% in patients with normal sinus rhythm or 6.4% of the entire Cohort) in patients with sinus rhythm following successful Cox-Maze procedure, and also demonstrated that restoration of sinus rhythm would not enough to avoid the stroke [8]. Furthermore, they concluded that the absence of left atrial mechanical contraction would be a strong independent risk factor for the postoperative stroke. The consideration is that a history of preoperative stroke is closely related with postoperative stroke regardless of left atrial mechanical contraction, albeit only univariate analysis which had been carried out. Meanwhile, the CHA_2_DS_2_-VASc score for stroke prediction [20] was analyzed as a risk factor. Univariate analysis showed that high CHA_2_DS_2_-VASc score (score ≥2) appeared not to be related with postoperative stroke (p = 0.08). This was consistent with the result of Buber and colleague [8]. This outcome was unexpected due to the fact that stroke risk score took account of history of stroke, transient ischemic attack and thromboembolism, which would be considered high risk in these cases. Such finding might have resulted from the aspect that the CHA_2_DS_2_-VASc score consisted of the combination of several risk factors.

In this study, all patients underwent left atrial ablation with cryoablation system. Left atrial ablation has the benefits of less procedure time, less procedure extent and less incidence of bradyarrhythmias. The left atrial ablation would be a more appropriated procedure than biatrial ablation with respect to a minimally invasive technique [21, 22]. Based on the fact that it is the procedure for patients with a mitral valve disease in which AF developed in the posterior wall of the left atrium and the periphery of pulmonary veins, it was judged that sufficient efficacy could be attained with the left-sided Cox Maze IV procedure. Only the left-sided procedure was carried out on the notion that, since the procedure could be performed in merely about 10 minutes, there would be no effect on morbidity or mortality by the surgery, and that there were reports of safe, simple and excellent results coming out of this procedure [10, 21]. This study revealed that overall success rate of Cox-Maze procedure was about 70% (Table 3). This was comparable to that of previous report in which patient underwent left-sided maze procedure with mitral valve surgery [21]. However this rate was lower than the biatrial maze with its rate showing 90% [5–7]. Compared with biatrial ablation, more frequent recurrence of atrial fibrillation in patients with mitral valve surgery [18] were evident in left atrial ablation. In this study, 139 patients (58%) had rheumatic mitral valve disease and 80 patients (33%) underwent concomitant aortic valve surgery. Previous studies demonstrated that patients with a rheumatic mitral valve disease and a multiple valvular disease have worse outcomes [16, 21, 23]. These reports supported the low success rate of the maze procedure among these patients with a valvular ailment.

**Table 3:** **Postoperative rhythm status**

	NSR	AF or A flat	Junctional rhythm	Pacing rhythm
3 months	71.1%	25.1%	2.1%	1.7%
6 months	70.9%	25.6%	1.7%	1.7%
1 year	73.4%	22.7%	2.1%	1.7%
2 years	72.2%	23.9%	1.7%	2.2%

*Abbreviations*: NSR, normal sinus rhythm; AF, atrial fibrillation; A flat, atrial flatter.

This study had a number of limitations. Having conducted a retrospective study with observational data, albeit all subjects were followed up for observations, these subjects were not homogenous and follow-up duration was relatively short. Only 142 of 240 patients underwent isolated mitral valve surgery including mitral valve repair, repair of function tricuspid regurgitation as well as Cox-Maze procedure, and others had surgery of double valve diseases or of the concomitant coronary artery surgery. This might have affected the success rate of the Cox-Maze procedure and the incidence rate of the stroke. A long-term study is necessary to confirm the results of this investigation. The results of this study were reported with rhythm statuses at intervals, and this might have overestimated the success rate of the Cox-Maze procedure [17]. A large scaled multi-center study is needed to overcome several limitations of which limited sample size and a single-center study.

## Conclusions

A history of preoperative stroke would increase the postoperative risk of stroke, following the Cox-Maze procedure concomitantly with mitral valve repair, in the group of patients with permanent AF accompanied by a structural mitral valve disease. Therefore, patients who had experienced ischemic strokes might require preventive anticoagulation even in cases with sustained sinus rhythm.
